# Molecular epidemiology of force of infection in malaria

**DOI:** 10.1186/1475-2875-9-S2-O14

**Published:** 2010-10-20

**Authors:** Ingrid Felger, Sonja Schoepflin, Benson Kiniboro, Peter A Zimmerman, Ivo Mueller

**Affiliations:** 1 Swiss Tropical and Public Health Institute, Socinstr. 57, 4002-Basel, Switzerland; 2 Papua New Guinea Institute of Medical Research, PO Box 60, Goroka, Eastern Highland Province 441, Papua New Guinea; 3 Center for Global Health and Diseases, Case Western Reserve University, Cleveland, OH 44106-7286, USA

## Background

Genotyping *Plasmodium falciparum* parasites in longitudinal studies provided a new option to estimate force of infection (FOI). FOI, defined as the number of new *P.falciparum* clones acquired over time, is a molecular parameter equally suitable for describing basic malaria epidemiology and measuring outcomes of clinical trials of antimalarial interventions. We investigated the ability of molecular parameters to explain differences in the risk of *P. falciparum* infection and disease between wet and dry seasons, among age groups and with respect to insecticide-treated mosquito net use.

## Methods

264 children between one and three years of age from Papua New Guinea were followed over 16 months with active detection of infection at two-monthly intervals and during episodes of febrile illness. A polymerase chain reaction (PCR) for the highly polymorphic genotyping marker merozoite surface antigen 2 was performed in all blood samples. To track individual parasite clones in consecutive blood samples with maximal resolution PCR fragments were sized by capillary electrophoresis.

## Results

FOI was moderately age-dependent with children under two acquiring less new *P. falciparum* clones than older ages. FOI was significantly correlated to incidence of episodes, irrespective of whether a parasite density cut off was applied or not. Seasonal variation was observed in FOI and thus risk of illness. FOI was significantly higher during the rainy season (7.47 children/year) than in the dry season (4.30 children/year, p < 0.001). Following the high transmission season the risk of clinical illness was significantly lower than after a period of low transmission.

## Conclusions

Our analyses suggest a central role of molecularly determined FOI for explaining differences in the burden of clinical *P. falciparum* malaria in our cohort. FOI almost completely explained spatial variation, age trends and effect of bed net use on incidence. Acquisition of new parasite clones seems to be a major factor for clinical illness in these children. This study highlights the suitability of a new parameter, molecular FOI, for understanding the epidemiology of clinical malaria in young children. We propose to apply the molecular determined parameter FOI for monitoring effects of malaria interventions.

